# Comparison of Performance of Various Calculated Low-Density Lipoprotein (LDL) Methods in the Indian Population: A Hospital-Based Study

**DOI:** 10.7759/cureus.62517

**Published:** 2024-06-17

**Authors:** Happy Chutia, Sungdirenla Jamir, Gautom Handique

**Affiliations:** 1 Biochemistry, North Eastern Indira Gandhi Regional Institute of Health and Medical Sciences, Shillong, IND; 2 Biochemistry, Bethany Hospital, Shillong, IND; 3 Biochemistry, Lakhimpur Medical College and Hospital, Lakhimpur, IND

**Keywords:** low-density lipoprotein cholesterol, sampson’s formula, martin-hopkin’s formula, friedwald formula, cardiovascular diseases

## Abstract

Introduction: In recent times, there has been a surge in proposed alternative approaches to computing low-density lipoprotein cholesterol (LDL-C), with a focus on enhancing precision, particularly within diverse demographic and clinical groups. Our aim is to assess the agreement, precision, and practicality of these methods compared to direct LDL-C measurements, with the goal of identifying the most effective approach for estimating LDL-C in the Indian context.

Methods: It is a retrospective analytical study. Lipid profile data were gathered from the laboratory and organized in Microsoft Excel for analysis. LDL-C was computed using three different methods: the Friedwald formula, the Martin-Hopkins formula, and Sampson's formula. These calculations were then compared with the direct method of LDL-C estimation in two distinct groups: when triglyceride (TG) levels were less than 400 mg/dL and when TG levels exceeded 400 mg/dL. Bland-Altman plots were generated, and concordance correlation coefficients (CCCs) were computed to determine the most suitable calculated method.

Results: Data from 1,776 participants were analysed and divided into two groups. In both Group 1 (TG < 400 mg/dL) and Group 2 (TG > 400 mg/dL) considering bias, limits of agreements, and correlation coefficient, as seen on the Bland-Altman and CCC, Martin-Hopkins equation was found to be performing better than Friedwald and Sampson’s equation.

Conclusion: In this study, the Martin-Hopkins formula appears to be the most appropriate choice for precise LDL-C level measurements and indicated improved accuracy and consistency in LDL-C measurements, especially in individuals with elevated TG levels. This underscores its importance in ensuring precise assessment and suitable clinical management.

## Introduction

Cardiovascular diseases (CVDs) remain one of the leading causes of mortality globally, with a significant burden in India. Among the various risk factors contributing to CVDs, elevated levels of low-density lipoprotein cholesterol (LDL-C) have been identified as a key modifiable risk factor and continue to be the primary target for treatment in addressing dyslipidaemia [1]. Accurate measurement of LDL-C is crucial for effective risk assessment and management strategies. Traditionally, LDL-C levels have been estimated using the Friedewald formula, which relies on measurements of total cholesterol, high-density lipoprotein cholesterol (HDL-C), and triglycerides (TGs). However, this method may not be as accurate in certain populations, particularly in individuals with high TG levels (>400 mg/dL), non-fasting samples, or those with metabolic abnormalities.

In recent years, several alternative methods for calculating LDL-C have been proposed, aiming to improve accuracy, especially in populations with diverse demographic and clinical characteristics. These methods incorporate various factors, such as non-HDL-C, apolipoprotein B, or direct measurement of LDL-C using specialized assays [2,3].

While several studies have scrutinized these alternative methods across different populations, there is a scarcity of data regarding their relative efficacy within the Indian demographic. While numerous studies have evaluated the performance of these alternative LDL-C calculation methods in various populations, limited data are available on their comparative effectiveness in the Indian population. Given the unique genetic, dietary, and lifestyle factors prevalent in India, it is essential to assess the suitability and accuracy of these methods in this demographic.

Therefore, this study aims to compare the performance of various calculated LDL methods in the Indian population. By evaluating the concordance, accuracy, and clinical utility of these methods against direct LDL-C measurements, we seek to provide insights into the optimal approach for LDL-C estimation in the Indian context. Such information is crucial for enhancing cardiovascular risk assessment, guiding personalized treatment strategies tailored to the needs of Indian patients, as well as to lower laboratory expenses [4].

## Materials and methods

We reviewed the records of blood samples that were sent to our Clinical Biochemistry Laboratory in a tertiary care institute in India for estimation of fasting lipid profile. The data of 1776 patients were collected after obtaining prior permission from the concerned authority, for a period of six months. The data encompassed a varied assortment of patients, some with dyslipidaemia and others without.

As a routine procedure, the samples were collected after 10-12 hours of overnight fasting by withdrawing 3 mL of venous blood in a plain vial. The samples were centrifuged at 3,000 rpm for 20 min to obtain serum and were analyzed for lipid profile on the same day.

Serum total cholesterol (TC) levels were assessed using the cholesterol oxidase-peroxidase method employing a commercial kit from Beckman Coulter (Indianapolis, IN) on the Beckman Coulter 5800 analyzer, demonstrating a coefficient of variation (CV) of 3% [5]. Serum TG levels were determined utilizing the glycerol-3 phosphate oxidase-3,5-dichloro-2-hydroxybenzenesulfonic acid method on the same analyzer, employing a commercial kit from Beckman Coulter with a CV of 3% [6]. High-density lipoprotein cholesterol (HDL-C) levels were assessed using a commercial kit from Beckman Coulter, employing a homogeneous method with a CV of 3%. This method utilizes a detergent that specifically solubilizes HDL lipoprotein particles, allowing HDL cholesterol to react with cholesterol esterase and cholesterol oxidase in the presence of chromogens to generate a color product [7].

Direct LDL-C estimation

The LDL-C test operates on a two-reagent homogeneous system, consisting of two distinct phases. In the initial phase, a unique detergent is employed to dissolve cholesterol from non-LDL lipoprotein particles. This dissolved cholesterol undergoes enzymatic reactions involving cholesterol esterase, cholesterol oxidase, peroxidase, and 4-aminoantipyrine, ultimately producing a colorless end product. In the subsequent phase, a second detergent in reagent 2 liberates cholesterol from LDL lipoproteins. This liberated cholesterol reacts with cholesterol esterase, cholesterol oxidase, and a chromogen system, resulting in the formation of a blue-colored complex measurable bichromatically at 540/660 nm. The observed increase in absorbance is directly correlated with the concentration of LDL-C in the sample [8].

LDL-C was calculated by the Friedwald equation by subtracting the concentration of cholesterol within all lipoproteins other than LDL from the concentration of TC. It was calculated by the following equation: LDL-C (mg/dL or mmol/L) = (Total cholesterol) - (HDL-C) - (Triglycerides)/5 [9].

LDL-C in the Martin-Hopkins formula was computed utilizing a Microsoft Excel spreadsheet acquired from Johns Hopkins Medicine, which replaces the fixed denominator with an adjustable factor X. This adjustable factor X changes following an empirically derived 180-cell strata based on the varying concentration of TG and non-HDL [10].

LDL-C in the Sampson formula was computed utilizing the following equation:

LDL-C = (Total cholesterol)/0.948 - (HDL-C)/0.971 - ((Triglycerides)/8.56 + (Triglycerides) × (non-HDL-C)/2140 - (Triglycerides)2 /16100) - 9.44 [11].

The total allowable limit of ±12%, which is recommended by the National Cholesterol Education Program Adult Treatment Panel III (NCEP ATP III) [12].

Statistical analysis

A database was constructed on Microsoft Excel 2019, and statistical analyses were done. Discrete data were reported as mean, standard deviation, and confidence range. The Bland-Altman plot was created using difference and average values between the estimated LDL and the three different methods of the calculated LDL and then compared. The concordance correlation coefficient was performed to find the level of agreement and consistency between the measurements of LDL by the direct method and the calculated methods.

## Results

**Figure 1:**
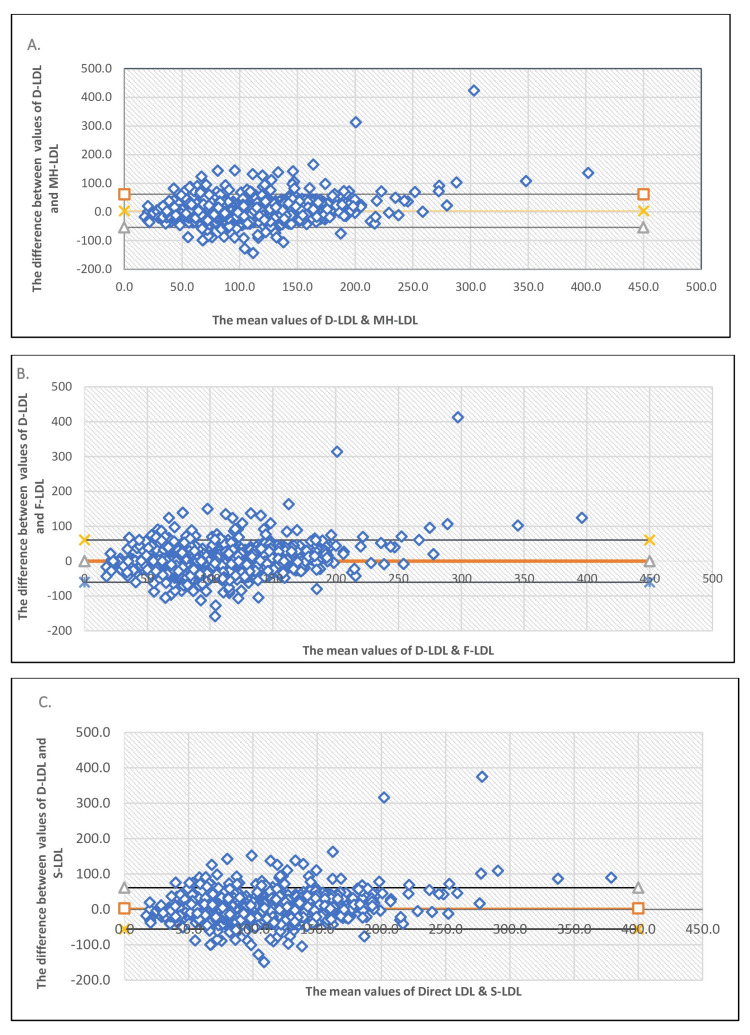
Bland-Altman (B & A) plots for Group 1. (A) Bland-Altman plot for Martin-Hopkins (MH)-LDL compared to D-LDL, for TG < 400 mg/dL. (B) Bland-Altman plot for Friedwald (F)-LDL compared to D-LDL, TG < 400 mg/dL. (C) Bland-Altman plot for Sampson (S)-LDL compared to D-LDL, TG < 400 mg/dL.

The data from 1,776 participants collected in a period of six months were included in the study. The Bland-Altman (B & A) plots for the different calculated LDL methods compared to direct LDL (D-LDL) estimation are shown in Figures 1A-1C when TG is less than 400 mg/dL (Group 1). The number of samples in this group is 1,718 (n).

Table 1 shows all the laboratory characteristics of the participants. The mean age of the participants is 46.5±14.2 years. The participants were divided into two groups depending on the value of triglyceride (TG): as Group 1 when TG < 400 mg/dL and Group 2 when high TG > 400 mg/dL.

**Table 1:** Showing the various parameters of the study groups. D-LDL, Direct low-density lipoprotein; F-LDL, Friedwald formula-low-density lipoprotein; HDL, high-density lipoprotein; LDL, low-density lipoprotein; MH-LDL, Martin-Hopkin equation-low-density lipoprotein; TC, total cholesterol; S-LDL, Sampson equation-low-density lipoprotein

Parameter	Group1 (n=1,718) TG < 400 mg/dL	Confidence range	Stdev	Group 2 (n=58) TG > 400 mg/dL	Confidence range	Stdev
	MEAN			MEAN		
TC (mg/dL)	168	175.56-170.22	56.5	217.5	234.88-200.12	66.1
HDL (mg/dL)	38	39.49-38.29	12.7	34.5	39.97-32.80	13.63
Non-HDL (mg/dL)	129	166.00-131.57	51.45	178.5	200.90-168.74	61.15
TG (mg/dL)	129	152.08-144.91	75.7	509.5	615.33-505.19	209.4
D-LDL (mg/dL)	104.33	106.17-102.50	38.78	118.5	132.09-109.78	42.4
F-LDL (mg/dL)	104.30	106.50-102.11	46.37	69.4	86.26-59.27	51.3
MH-LDL (mg/dL)	108.33	110.50-106.16	45.87	110.5	125.36-102.70	43.08
S-LDL (mg/dL)	107.1	109.29-104.95	45.86	88.25	101.86-81.10	39.45

Figure 1A shows the Bland-Altman (B & A) plot in which the X-axis represents the mean of direct LDL-C and Martin-Hopkins LDL-C (MH-LDL) and the Y-axis represents the difference between the values of direct LDL-C and MH-LDL. By this plot bias between the mean differences, agreement interval estimates were done, within which 95% of the differences of the MH-LDL fall, compared to direct LDL-C. Similarly, Figures 1B-1C are the B&A plots for LDL calculated as per the Friedwald formula (F-LDL) and Sampson formula (S-LDL), respectively, compared to direct-LDL estimation.

The bias, standard deviation of the difference of the mean, and the upper and lower limits of agreements (LoA) have been shown in Table 2 for various calculated methods compared to direct LDL-C. It is seen that LDL calculated as per F-LDL has the smallest bias (0.09), but its LoA range is relatively wide (-60.51 to 60.69) compared to MH-LDL (-53.72 to 61.92) and S-LDL (-55.55 to 61.36). MH-LDL and S-LDL have slightly higher biases but narrower LoA ranges. When we looked for concordance correlation coefficient (CCC), it was found that D-LDL-C showed the strongest agreement with the MH-LDL method (CCC=0.817), followed by a moderate agreement with the other two methods, that is, F-LDL (CCC=0.73) and S-LDL(CCC=0.63).

**Table 2:** Bias and standard deviation of the difference of mean and upper and Lower limits of agreement for Group 1. F-LDL, Friedwald formula-low-density lipoprotein; MH-LDL, Martin-Hopkin equation-low-density lipoprotein; S-LDL, Sampson equation-low-density lipoprotein

Method	Bias	Standard deviation of the difference of mean	Upper LoA	Lower LoA
MH-LDL	4.1	29.50	61.92	-53.72
F-LDL	0.09	30.92	60.69	-60.51
S-LDL	2.9	29.82	61.36	-55.55

**Figure 2:**
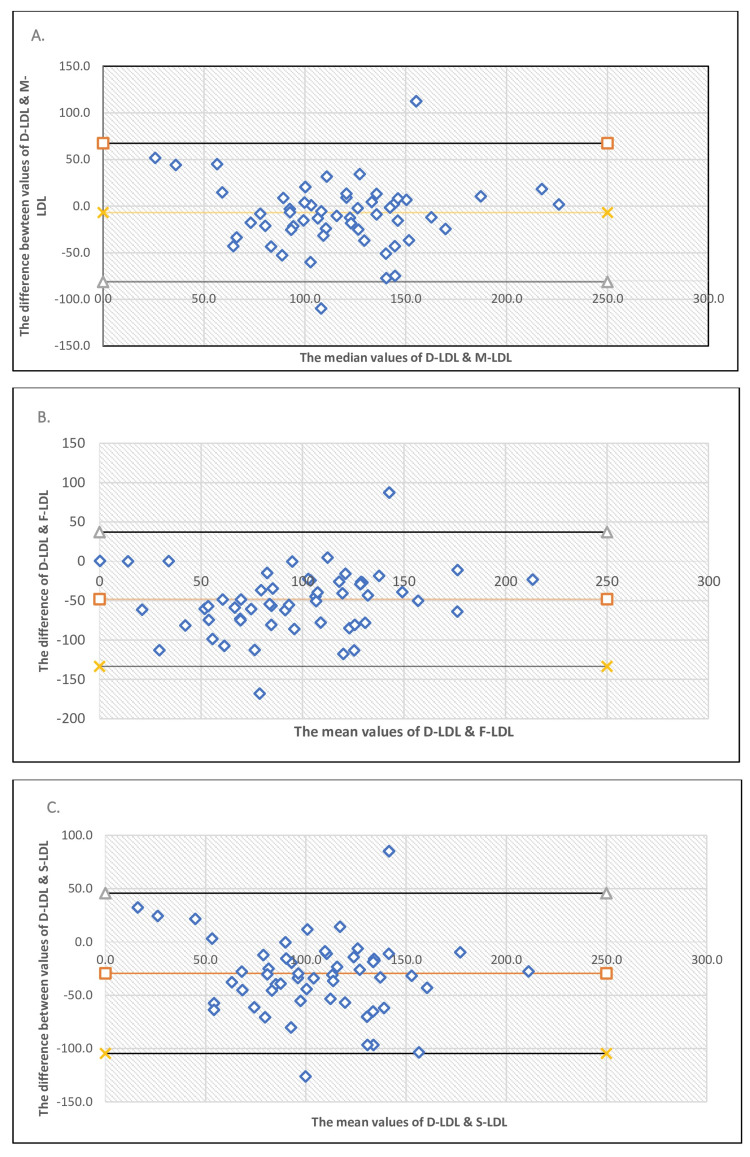
Bland-Altman (B & A) plot for Group 2. (A) Bland-Altman plot for MH-LDL compared to D-LDL, TG > 400 mg/dL. (B) Bland-Altman plot for F-LDL compared to D-LDL, TG > 400 mg/dL. (C) Bland-Altman plot for S-LDL compared to D-LDL, for TG > 400 mg/dL.

The B & A plots for the different calculated LDL methods compared to D-LDL estimation are shown in Figures 2A-2C when TG is more than 400 mg/dL. The number of samples in Group 2 is 58 (n). By these plots’ bias between the mean differences, agreement interval estimates were done, within which 95% of the differences of the various calculated LDL methods fall, compared to D-LDL-C.

The bias, standard deviation of the difference of the mean, and the upper and lower LoA have been shown in Table 3 for various calculated methods compared to D-LDL-C for Group 3. From Figures 2A-2C and Table 3, it has been found that MH-LDL has the smallest bias (-6.9), compared to F-LDL (-48.16) and S-LDL (-29.5), and its LoA range is relatively narrow (-81.18 to 67.38) compared to those of F-LDL (-133.48 to 37.15) and S-LDL (-104.61 to 45.69). When we looked for the CCC, it was found that D-LDL showed the strongest agreement with the MH-LDL method (CCC=0.76), followed by a moderate agreement with the other two methods, that is, S-LDL (CCC=0.44) and F-LDL (CCC=0.45).

**Table 3:** Bias and standard deviation of the difference of mean and upper & lower limits of agreement for Group 2. F-LDL, Friedwald formula-low-density lipoprotein; LoA, limits of agreements; MH-LDL, Martin-Hopkin equation-low-density lipoprotein; S-LDL, Sampson equation-low-density lipoprotein

Method	Bias	Standard deviation of the difference of mean	Upper LoA	Lower LoA
MH-LDL	-6.9	37.90	67.38	-81.18
F-LDL	-48.16	43.53	37.15	-133.48
S-LDL	-29.5	38.34	45.69	-104.61

## Discussion

A total of 1,776 participants’ lipid profiles were analysed and divided into two groups based on TG: Group 1 when TG < 400 mg/dL and Group 2 when TG > 400 mg/dL. From the Bland-Altman analysis in Group 1, it has been found that F-LDL gives comparatively lesser bias (0.09%) than the other two methods, that is, the Martin-Hopkins formula (4.1%) and Sampson’s formula (2.9%). However, it has a wider limit of agreements. The total allowable bias is ±12% as recommended by the NCEP ATP III. None of the methods exceed this threshold, suggesting that they are all within an acceptable range of bias according to this guideline. However, when we look into the CCC, the Martin-Hopkins formula is in stronger agreement (CCC=8.7) than the Friedwald formula (CCC=0.73) and Sampson's formula (CCC=0.63). Considering both analyses, it can be inferred that, when TGs are below 400 mg/dL, the Martin-Hopkins formula may be considered preferable for calculating LDL levels.

In Group 2, from Bland-Altman analysis, it has been found that the Martin-Hopkins formula exhibits the smallest bias (-6.9) compared to the Friedwald formula (-48.16) and Sampson’s formula (-29.5) when TG levels are above 400 mg/dL, which is within allowable error (±12%). Additionally, the LoA range for the Martin-Hopkins formula is relatively narrow compared to the other two formulae. When assessing the agreement between methods using CCC, the strongest agreement is observed for the Martin-Hopkins formula (CCC=0.76), compared to the Friedwald formula (0.45) and Sampson’s formula (0.44). This indicates that MH-LDL may provide more accurate LDL-C measurements in when TG is more than 400 mg/dL. The Martin-Hopkins formula was also found more accurate by Cartier et al. in their study [13]. In a study conducted by Briers et al., they assessed the agreement between the Friedwald, Sampson's, and Martin-Hopkins formula and concluded that the Martin-Hopkins formula was the most suitable option [14]. Similar findings were also found in a study done on familial hypertriglyceridemia by Mehta et al. in Mexico [15].

Benefits of the alternative calculated method of LDL estimation

Cost-effectiveness: Calculated methods typically require fewer resources and equipment compared to direct methods, resulting in lower overall costs.

Accessibility: Calculated methods are often more accessible since they rely on readily available data such as lipid profiles.

Efficiency: Calculated methods can be faster and more efficient, allowing for quicker assessment of LDL levels and facilitating timely decision-making in clinical settings.

Reduced dependence on specialized equipment: Calculated methods may not require specialized equipment or reagents, making them more feasible for smaller clinics or remote areas where access to such resources may be limited.

Consistency: Calculated methods can provide consistent results over time, reducing variability compared to direct methods. A more precise assessment of LDL cholesterol levels, especially in cases where TG levels are high (>400 mg/dL) or when the patient has abnormal lipoprotein profiles, is particularly important where accurate LDL measurement is crucial for risk assessment and treatment decisions.

Limitations of the study

The Martin-Hopkins and other calculated formulae were developed primarily for use with fasting blood samples, and their accuracy may be affected in non-fasting samples, as TG levels can significantly affect the calculation. This was not seen in the study. The number of samples in Group 2 (i.e. TG > 400 mg/dL) was limited and therefore may not be sufficient to come to a fixed conclusion.

## Conclusions

The Martin-Hopkins formula emerges as the most promising option for accurately measuring LDL-C levels. Its smallest bias and relatively narrow LoA compared to the Friedwald and Sampson formulae suggest enhanced accuracy and consistency in LDL-C measurements, particularly in patients with elevated triglyceride levels, to ensure accurate assessment and appropriate clinical management. Despite being more intricate than the Friedwald formula, the Martin-Hopkin formula is adaptable to contemporary laboratory information systems. Furthermore, the absence of intellectual property constraints enables laboratories to adopt it freely, thereby enhancing the accuracy of LDL-C measurements. Direct methods are accurate and precise for the estimation of LD, but when LDL is to be frequently measured in the monitoring of dyslipidemia and/or atherosclerotic cardiovascular disease, it adds to the cost of treatment.

## References

[1] PS Jellinger, DA Smith, AE Mehta (2012). American Association of Clinical Endocrinologists' guidelines for management of dyslipidemia and prevention of atherosclerosis: executive summary. Endocr Pract.

[2] Aggarwal DJ, Kathariya MG, Verma DP (2021). LDL-C, NON-HDL-C and APO-B for cardiovascular risk assessment: looking for the ideal marker. Indian Heart J.

[3] Gupta R, Rao RS, Misra A, Sharma SK (2017). Recent trends in epidemiology of dyslipidemias in India. Indian Heart J.

[4] Lim HY, Burrell LM, Brook R, Nandurkar HH, Donnan G, Ho P (2022). The need for individualized risk assessment in cardiovascular disease. J Pers Med.

[5] Allain CC, Poon LS, Chan CS, Richmond W, Fu PC (1974). Enzymatic determination of total serum cholesterol. Clin Chem.

[6] Bucolo G, David H (1973). Quantitative determination of serum triglycerides by the use of enzymes. Clin Chem.

[7] Warnick GR, Benderson J, Albers JJ (1982). Dextran sulfate-Mg2+precipitation procedure for quantitation of high-density-lipoprotein cholesterol. Clin Chem.

[8] Harris N, Galpchian V, Thomas J, Iannotti E, Law T, Rifai N (1997). Three generations of high-density lipoprotein cholesterol assays compared with ultracentrifugation/dextran sulfate-Mg2+method. Clin Chem.

[9] Friedewald WT, Levy RI, Fredrickson DS (1972). Estimation of the concentration of low-density lipoprotein cholesterol in plasma, without use of the preparative ultracentrifuge. Clin Chem.

[10] Martin SS, Blaha MJ, Elshazly MB, Toth PP, Kwiterovich PO, Blumenthal RS, Jones SR (2013). Comparison of a novel method vs the Friedewald equation for estimating low-density lipoprotein cholesterol levels from the standard lipid profile. JAMA.

[11] Sampson M, Ling C, Sun Q (2020). A new equation for calculation of low-density lipoprotein cholesterol in patients with normolipidemia and/or hypertriglyceridemia. JAMA Cardiol.

[12] National Cholesterol Education Program (NCEP) Expert Panel on Detection, Evaluation Evaluation, and Treatment of High Blood Cholesterol in Adults (Adult Treatment Panel III) (2002). Third report of the National Cholesterol Education Program (NCEP) Expert Panel on Detection, Evaluation, and Treatment of High Blood Cholesterol in Adults (Adult Treatment Panel III) final report. Circulation.

[13] Cartier LJ, St-Coeur S, Robin A, Lagace M, Douville P (2020). Impact of the Martin/Hopkins modified equation for estimating LDL-C on lipid target attainment in a high risk patient population. Clin Biochem.

[14] Briers PJ, Langlois MR (2022). Concordance of apolipoprotein B concentration with the Friedewald, Martin-Hopkins, and Sampson formulas for calculating LDL cholesterol. Biochem Med (Zagreb).

[15] Mehta R, Reyes-Rodríguez E, Bello-Chavolla O, Guerrero-Díaz AC, Vargas-Vázquez A, Cruz-Bautista I, Aguilar-Salinas C (2018). Performance of LDL-C calculated with Martin's formula compared to the Friedewald equation in familial combined hyperlipidemia. Atherosclerosis.

